# Enhancement of the Antioxidant, Anti-Tyrosinase, and Anti-Hyaluronidase Activity of *Morus alba* L. Leaf Extract by Pulsed Electric Field Extraction

**DOI:** 10.3390/molecules25092212

**Published:** 2020-05-08

**Authors:** Wantida Chaiyana, Jakkapan Sirithunyalug, Suvimol Somwongin, Chanun Punyoyai, Natnaree Laothaweerungsawat, Pachabadee Marsup, Waranya Neimkhum, Artit Yawootti

**Affiliations:** 1 Department of Pharmaceutical Sciences, Faculty of Pharmacy, Chiang Mai University, Chiang Mai 50200, Thailand; jakkapan.s@cmu.ac.th (J.S.); suvimol_ampoo@hotmail.com (S.S.); spycooler_new@hotmail.com (C.P.); natnaree.la@gmail.com (N.L.); pch.marsup@gmail.com (P.M.); 2 Research Center of Pharmaceutical Nanotechnology, Chiang Mai University, Chiang Mai 50200, Thailand; 3 Department of Pharmaceutical Technology, Faculty of Pharmaceutical Sciences, Huachiew Chalermprakiet University, Samutprakarn 10250, Thailand; waranya.ne@gmail.com; 4 Department of Electrical Engineering, Faculty of Engineering, Rajamangala University of Technology Lanna, Chiang Mai 50300, Thailand; yartit@rmutl.ac.th

**Keywords:** *Morus alba*, pulsed electric field, antioxidant, anti-tyrosinase, anti-hyaluronidase, anti-aging

## Abstract

In this study we aimed to compare the chemical composition and biological activity between *Morus alba* L. leaf extract obtained with 95% *v*/*v* ethanol using a pulsed electric field (PEF) and the conventional maceration method. Extracts of *M. alba* leaves collected from Chiang Mai (CM), Sakon Nakon (SK), and Buriram (BR), Thailand, were investigated for 1-deoxynojirimycin content by high-performance liquid chromatography and for total phenolic content by the Folin–Ciocalteu method. Antioxidant activity was investigated by 2,2′-diphenyl-1-picrylhydrazyl (DPPH), 2,2’-azinobis-3-ethylbenzothiazoline-6-sulphonate (ABTS), and ferric reducing antioxidant power (FRAP) assay. Anti-tyrosinase and anti-hyaluronidase activity was investigated by in vitro spectrophotometry. The results show that this is the first study to indicate PEF as a novel method for enhancing the phenolic content and antioxidant, anti-tyrosinase, and anti-hyaluronidase activity of *M. alba* leaf extract (*P* < 0.05). PEF extract of *M. alba* leaves collected from BR had comparable ABTS^•+^ scavenging activity to l-ascorbic acid and comparable anti-tyrosinase activity to kojic acid (*P* > 0.05). On the other hand, PEF extract of *M. alba* leaves collected from SK exhibited significantly high anti-hyaluronidase activity, comparable to that of oleanolic acid (*P* > 0.05). Therefore, PEF is suggested for further *M. alba* leaf extraction in the production of natural whitening and anti-aging cosmetic ingredients.

## 1. Introduction

Mulberry (*Morus alba* L.), belonging to the genus *Morus* in the family Moraceae, is widely distributed in various climatic conditions ranging from tropical to temperate [1]. The fruit of *M. alba* is edible and widely used in the preparation of juices, smoothies, desserts, jams, etc. Additionally, *M. alba* fruit has a long history of being used as a medicinal food in China to prevent premature graying of hair; nourish the blood and kidneys; and treat fatigue, weakness, dizziness, constipation, urinary incontinence, tinnitus, and anemia [2,3]. Various active biological components of *M. alba* fruit have been previously reported, including anthocyanins, rutin, quercetin, chlorogenic acid, and polysaccharides [1]. Other than the fruit, the leaf part has also been utilized, especially for feeding silkworms in Asian countries [4]. Moreover, *M. alba* leaf has been reported for its protection against age-related behavioral and biochemical changes, since it is rich in 1-deoxynojirimycin, an inhibitor of α-glucosidase, which can alter cell surface glycosylation and plays an important role in protein turnover, tissue remodeling, diabetes, and skin aging [5,6,7]. Furthermore, cosmeceutical applications of *M. alba* leaf have also been proposed, since it possesses anti-aging, antioxidant, and anti-tyrosinase activity [8,9,10]. Therefore, developing an extraction technique that could enhance this cosmeceutical-related biological activity is attractive and worthwhile.

Pulsed electric field (PEF) is an application of high-voltage electric fields for brief periods of time into plant materials placed between two electrodes [11,12,13]. PEF promotes the modification of membrane permeability, known as electroporation or electropermeabilization, by which the mass transfer during extraction is enhanced [12,13]. PEF enhances a release of intracellular compounds from plant tissues and leads to increased extraction yield [12,13]. Additionally, PEF extraction has been reported as an economical process due to its relatively small power consumption [14]. PEF has been used for extraction of various natural compounds. Pretreatment with PEF has been reported to significantly increase the efficiency of betalain extraction from beetroot (*P* < 0.05) [11]. Additionally, a low pulse duration with a high pulse interval of PEF has been reported to increase the extraction efficiency of dry *Moringa oleifera* leaves [15]. PEF-assisted maceration of red grape juice also promoted wine color quality and the polyphenolic profile [16]. PEF also enhanced the extractability of carotenoids from various microalgae, including *Chlorella vulgaris*, *Spirulina platensis*, and *Nannochloropsis* sp. [17,18,19,20,21,22]. Furthermore, PEF has been defined as a green extraction technology because of its substantial minimization of toxic solvents, reduction of waste products, and lower energy consumption compared to conventional thermal extraction and dehydration processes [23,24,25].

Since PEF is an attractive method for extracting bioactive compounds from natural sources, the present study aimed to extract 1-deoxynojirimycin and phenolic compounds from *M. alba* leaves with 95% *v*/*v* ethanol using PEF extraction as cosmeceutical ingredients for topical application. In addition, the chemical composition and biological activity of *M. alba* leaf extract obtained by PEF were investigated in comparison with extract obtained by a conventional maceration method.

## 2. Results and Discussion

### 2.1. M. alba Leaf Extracts

**Figure 1:**
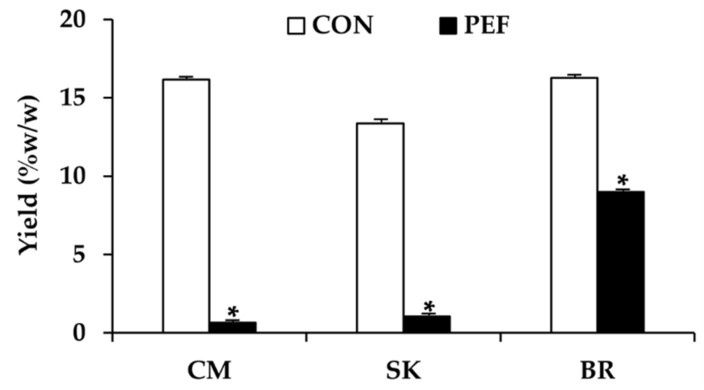
Yield of *Morus alba* leaf extract from Chiang Mai (CM), Sakon Nakon (SK), and Buriram (BR) when extracted using conventional maceration (CON) and pulsed electric field extraction (PEF). Asterisk (*) denotes significant difference between CON and PEF, *P* < 0.05.

The yield of *M. alba* leaf extract is shown in Figure 1. PEF yielded remarkably lower extract content than conventional maceration, due to different extraction duration and numbers of extraction cycles. It has been previously reported that an application of 300–1000 V/cm electric field strength and 100–1000 μs pulse duration was efficient to allow permeabilization of plant tissue and usually improved the extraction yield [14,26]. However, in the present study, maceration was performed over 24 h for 3 cycles, while PEF was employed for only 20 min once. Therefore, longer extraction duration and more extraction cycles led to higher content yield since PEF generates electroporation, which leads to electropermeabilization and enhances mass transfer during extraction [9,10].

### 2.2. Chemical Composition of M. alba Leaf Extracts

**Figure 2:**
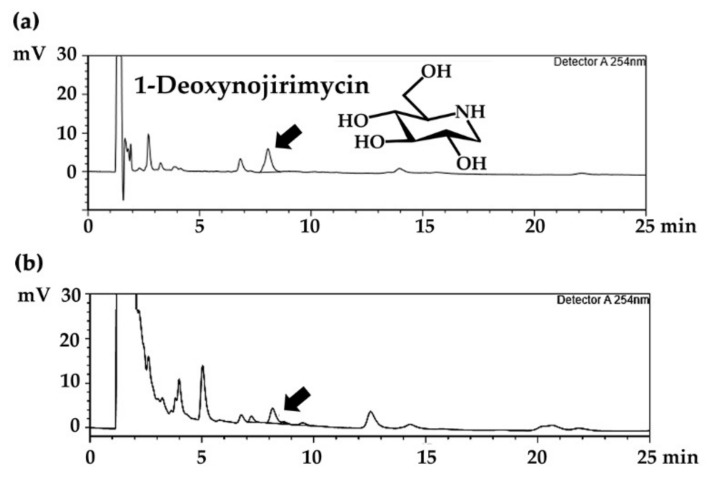
HPLC chromatograms of (**a**) 1-deoxynojirimycin and (**b**) *M. alba* leaf extract.

**Figure 3:**
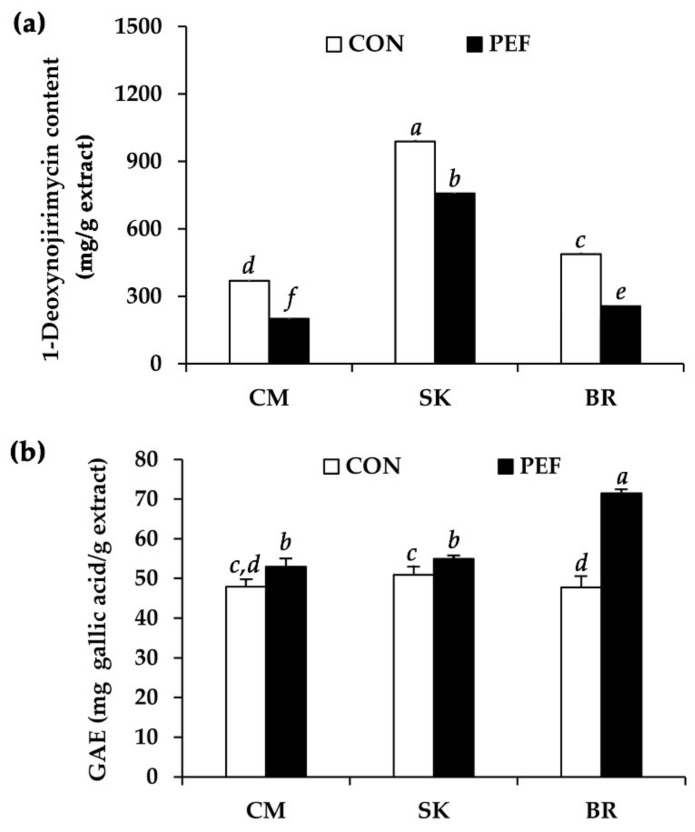
(**a**) 1-Deoxynojirimycin content and (**b**) total phenolic content, expressed as gallic acid equivalent (GAE), of *M. alba* leaf extract from Chiang Mai (CM), Sakon Nakhon (SK), and Buriram (BR). *a*, *b*, *c*, *d*, *e*, *f* denote significant differences among samples in each experiment, *P* < 0.05.

*M. alba* leaf extract was investigated for 1-deoxynojirimycin and total phenolic content. HPLC chromatograms of 1-deoxynojirimycin and *M. alba* leaf extract are shown in Figure 2. A peak detected at 8.105 min on a chromatogram was in accordance with the 1-deoxynojirimycin peak detected at 8.064 min. Internal standardization was already performed to confirm the 1-deoxynojirimycin peak. However, 1-deoxynojirimycin is not a major component of *M. alba* leaf extract, since it presented less than 1000 mg/g extract in all samples, as shown in Figure 3a. The conventional maceration method could extract significantly higher amounts of 1-deoxynojirimycin than PEF in all *M. alba* leaf extracts (*P* < 0.05). Therefore, PEF was not useful for extraction. Among various sources of *M. alba* leaves, SK yielded the highest 1-deoxynojirimycin content (989.9 ± 4.5 mg/g extract), followed by BR (369.9 ± 0.2 mg/g extract) and CM (575.9 ± 3.1 mg/g extract). The 1-deoxynojirimycin content investigated in the present study related well with a previous report noting that most collected *M. alba* leaf samples contained 0.04–0.06% of 1-deoxynojirimycin content (equivalent to 400–600 mg/g extract) [27]. Various factors have been reported to affect the 1-deoxynojirimycin content in *M. alba* leaves, e.g., maturity of leaves, period of collection, species, and place of origin [27,28]. Since these factors were the same in the present study, place of origin might be a factor that affects the chemical composition and biological activity of *M. alba* leaf extract. The likely explanation is that it might be due to variations in climate, geography, and soil quality in each region. The climate of each region was a bit different. The annual temperature in CM (mean 26 °C, min 14 °C, max 39 °C) was lower than that in BR (mean 27 °C, min 22 °C, max 32 °C) and SK (mean 27 °C, min 15 °C, max 39 °C) [29,30,31,32]. Additionally, most of BR and SK is plateau tablelands, whereas CM is mostly mountains and groves. On the other hand, there are volcanic soils, which are suitable for agriculture, in some regions of BR.

In contrast to 1-deoxynojirimycin content, PEF could extract significantly higher phenolic content from *M. alba* leaves compared to the conventional maceration method, as shown in Figure 3b, although the extraction duration was much shorter. Therefore, PEF was suggested as a high efficiency method for extraction of phenolic compounds. It was remarked that only 20 min of PEF extraction could be compared to 3 days of conventional maceration. *M. alba* leaves from BR extracted by PEF had the highest phenolic content (71.5 ± 0.9 mg gallic acid/g extract), followed by SK (54.9 ± 0.4 mg gallic acid/g extract) and CM (53.9 ± 2.1 mg gallic acid/g extract). The high phenolic content of PEF extract of *M. alba* leaves from BR could support its remarkably higher yields very well. Therefore, it could be seen that more extracted phenolic compounds resulted in higher yield. Additionally, PEF was previously reported to enhance phenolic concentration in extracts of orange [33], grape [34], sorghum [35], apple [35], onion [36], spearmint [37], etc. According to the non-thermal technology of PEF, it causes less degradation of chemical compounds in natural products compared to the conventional thermal process hence is used as an alternative to prevent thermolabile compound degradation during dehydration processes [38,39]. However, high voltage of PEF has been reported to increase degradation of some natural products, including phenolics. As phenolics donate electrons to other compounds, they respond to the alternate electric field [40]. Nevertheless, degradation was detected only by the thermal effect [40]. Therefore, suitable conditions of PEF would result in enhanced extraction efficiency without degradation of biological compounds.

PEF was more suitable for extraction of phenolic compounds than 1-deoxynojirimycin, which has been widely known as an α-glucosidase inhibitor used for diabetes mellitus [41]. PEF extracts were suitable for further development as cosmeceutical products rather than nutraceutical products since phenolics have been widely used in the cosmetics industry [42].

### 2.3. Antioxidant Activity of M. alba Leaf Extract

**Figure 4:**
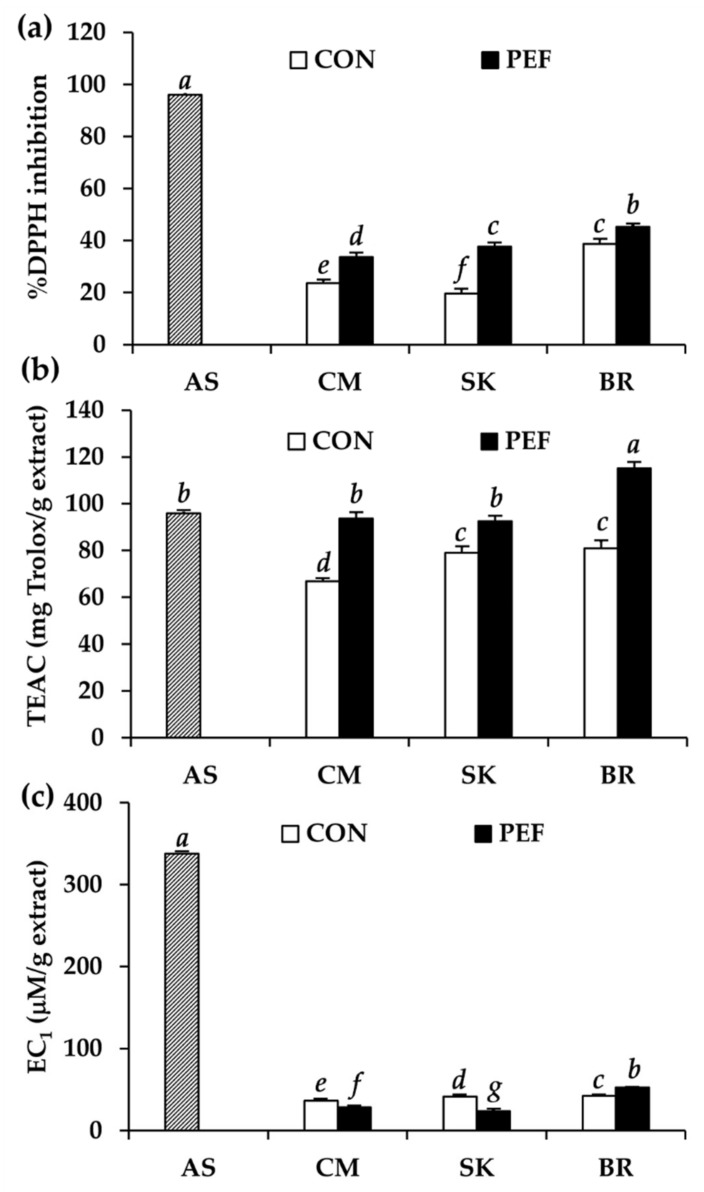
(**a**) DPPH^•^ inhibition, (**b**) ABTS^•+^ inhibition, and (**c**) ferric reducing antioxidant power of ascorbic acid (AS) and *M. alba* leaf extract from Chiang Mai (CM), Sakon Nakhon (SK), and Buriram (BR) expressed as % inhibition, Trolox equivalent antioxidant capacity (TEAC), and equivalent concentration (EC_1_). *a*, *b*, *c*, *d*, *e*, *f*, *g* denote significant differences among samples in each experiment, *P* < 0.05.

Antioxidant activity of *M. alba* leaf extract is shown in Figure 4. All extract obtained by PEF had significantly higher radical scavenging activity compared to the conventional maceration method (*P* < 0.05). On the other hand, only *M. alba* leaves from BR had significantly higher ferric reducing antioxidant power (*P* < 0.05). Among different extracts, the PEF extract from BR had the highest inhibitory activity against DPPH^•^ and ABTS^•+^ and the highest ferric reducing antioxidant power with DPPH^•^ inhibition of 45.3 ± 0.8%, Trolox equivalent antioxidant capacity (TEAC) value of 115.1 ± 2.8 mg Trolox/g extract, and EC_1_ value of 52.4 ± 0.5 mg FeSO_4_/g extract (*P* < 0.05). The likely explanation might be the high phenolic content, since phenolics have been shown to have potent antioxidant activity and are usually responsible for the antioxidant activity of several natural extracts [43].

Interestingly, all PEF extracts had comparable or even higher ABTS^•+^ scavenging activity compared to ascorbic acid, a commonly used antioxidant in the food, pharmaceutical, and cosmetic industries (*P* > 0.05). Therefore, PEF extract, especially from BR, was suggested as a potent natural antioxidant via the radical scavenging mechanism. Excessive production of reactive oxygen species (ROS) in the human body leads to oxidative stress, resulting in cell damage and cell death [44]. In addition, oxidative stress occurring in the skin plays a major role in the aging process and leads to wrinkles [45]. Therefore, radical scavengers, which have the ability to sacrificially reduce ROS formation and break down the oxidative chain reaction, finally prevent biomolecular damage [46,47]. Therefore, antioxidants have been widely used in cosmetic products as anti-aging components [48,49]. PEF extract from BR, which had the most potent radical scavenging activity, would be an attractive natural anti-aging component in further cosmetic/cosmeceutical product development.

### 2.4. Anti-Tyrosinase Activity of M. alba Leaf Extract

**Figure 5:**
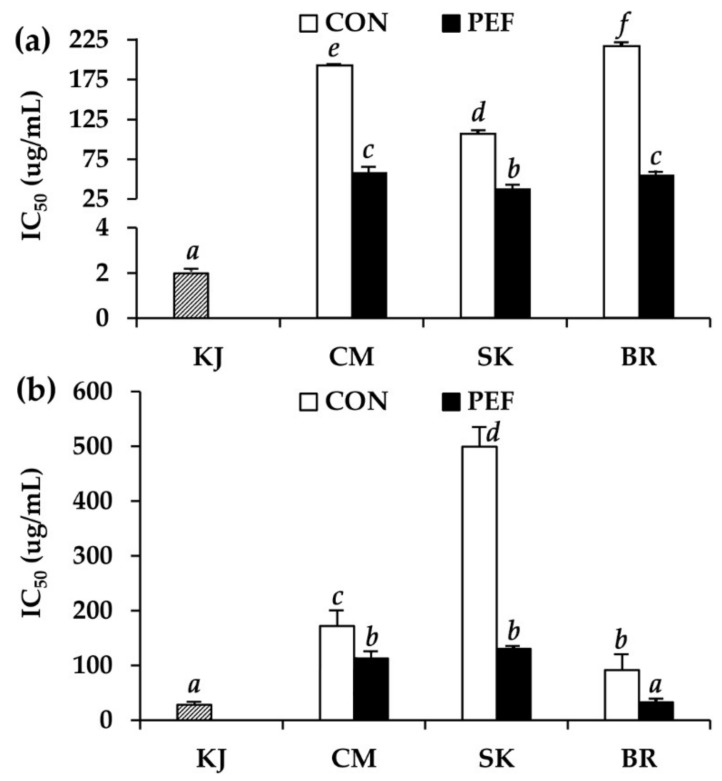
IC_50_ against tyrosinase with substrates (**a**) l-tyrosine and (**b**) l-DOPA of kojic acid (KJ) *M. alba* leaf extract from Chiang Mai (CM), Sakon Nakhon (SK), and Buriram (BR). *a*, *b*, *c*, *d*, *e*, *f* denote significant differences among samples in each experiment, *P* < 0.05.

The anti-tyrosinase activity of *M. alba* leaf extract is shown in Figure 5. Since tyrosinase plays a key role in melanin biosynthesis, natural extracts that have an inhibitory effect on melanin formation would be a good choice for the cosmetic purpose of whitening the skin [50]. All PEF extracts had a significantly more potent whitening effect than conventional crude extracts (*P* < 0.05). Although *M. alba* leaves have previously been shown to have a strong tyrosinase inhibitory effect, with inhibition comparable to arbutin [51], the present study is the first to highlight PEF as a novel extraction method to enhance anti-tyrosinase activity. Among different *M. alba* leaf extracts, PEF extract from BR exhibited the highest anti-tyrosinase activity, with IC_50_ values against tyrosinase activity on l-tyrosine and l-DOPA of 54.1 ± 5.4 and 32.2 ± 3.4 μg/mL, respectively (*P* < 0.05). Interestingly, PEF extract from BR exhibited comparable anti-tyrosinase activity to kojic acid, with an IC_50_ value of 28.0 ± 5.1 μg/mL when the substrate was l-DOPA (*P* > 0.05). Polyphenols have been reported as components responsible for depigmentation properties of *M. alba* leaves [50]. Therefore, it can be noted that PEF is an extraction method capable of enhancing phenolic extraction from *M. alba* leaves, resulting in an extract with a potent whitening effect.

### 2.5. Anti-Hyaluronidase Activity of M. alba Leaf Extract

Hyaluronic acid (also called hyaluronan) is an essential component of the extracellular matrix of the skin that has some elastic properties [51]. However, it can be degraded into smaller fragments by hyaluronidases, which hydrolyze the disaccharides at the hexosaminidic β (1–4) linkages, and the progressive loss of dermal hyaluronic acid is one of the hallmarks of skin aging [51]. Inhibition of hyaluronidase activity would thus be another way to retard the skin aging process.

**Figure 6:**
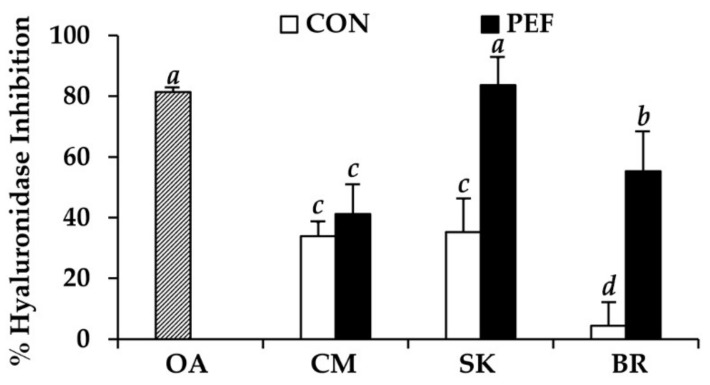
Hyaluronidase inhibitory activity of oleanolic acid (OA) and *M. alba* leaf extract from Chiang Mai (CM), Sakon Nakhon (SK), and Buriram (BR). *a*, *b*, *c*, *d* denote significant differences among samples in each experiment, *P* < 0.05.

The anti-hyaluronidase activity of *M. alba* leaf extract is shown in Figure 6. Conventional crude *M. alba* leaf extract demonstrated mild to moderate anti-hyaluronidase activity. PEF extraction significantly enhanced the anti-hyaluronidase activity of *M. alba* leaf extract. Interestingly, PEF extract from SK showed the highest anti-hyaluronidase activity (inhibition = 83.6 ± 9.1%), which was comparable to that of oleanolic acid (inhibition = 81.3 ± 2.9%) (*P* > 0.05). Therefore, *M. alba* leaf extract, which potentially inhibits hyaluronidase, would be an attractive natural anti-aging agent.

## 3. Materials and Methods

### 3.1. Plant Materials

**Figure 7:**
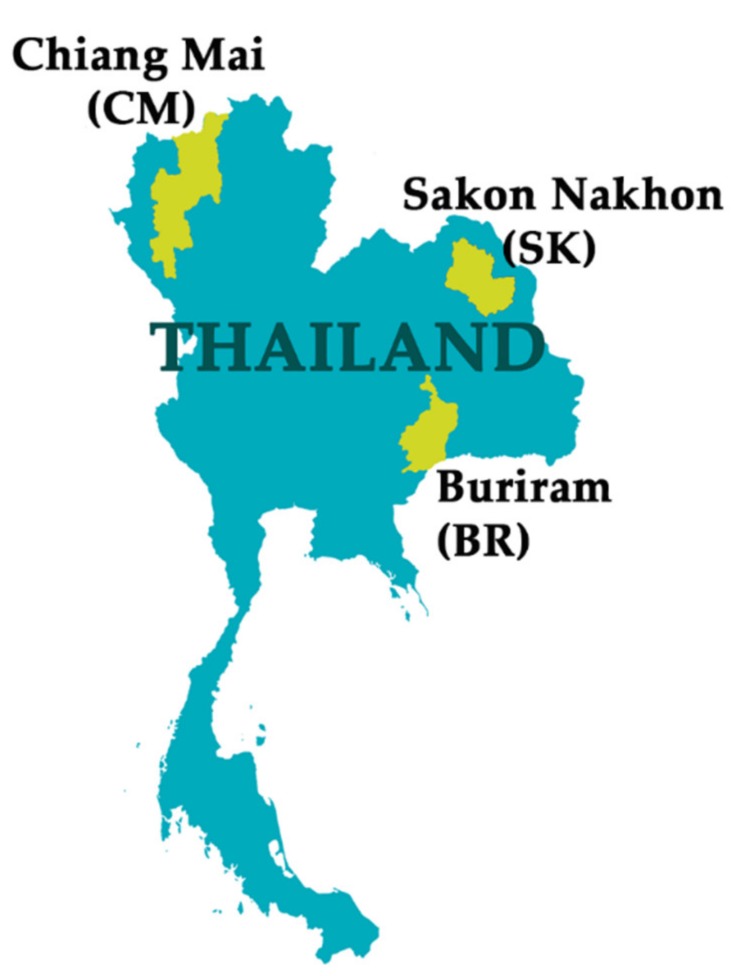
Source locations for *M. alba* leaves: Chiang Mai (CM), Sakon Nakon (SK), and Buriram (BR).

Fresh, fully matured *M. alba* leaves were obtained during October 2018 from local farms in CM (northern region of Thailand), SK (upper northeastern region), and BR (lower northeastern region), as shown in Figure 7. The fresh leaves were washed with tap water to remove dirt and allowed to dry at room temperature overnight. Subsequently, 200 g of *M. alba* leaves from each location were further dried in an oven (UF110, Memert, Germany) at 40 °C. The dried *M. alba* leaves were then ground into fine powder using a Moulinex DB81 blender (Moulinex, Paris, France) and kept in well-closed containers until further extraction.

### 3.2. Chemical Materials

Tyrosinase from mushroom lyophilized powder (EC 1.14.18.1), hyaluronidase from bovine testes (EC 3.2.1.35), gallic acid, kojic acid, Trolox, l-ascorbic acid, l-tyrosine, l-DOPA, trifluoroacetic acid, Folin–Ciocalteu reagent, 2,4,6 tripyridyl-s-triazine (TPTZ), 2,2′-diphenyl-1-picrylhydrazyl-hydrate (DPPH), and 2,2’-azinobis 3-ethylbenzothiazoline-6-sulphonate (ABTS) were purchased from Sigma-Aldrich (St. Louis, MO, USA). Ferrous sulfate (FeSO_4_), sodium carbonate (Na_2_CO_3_), sodium acetate (CH_3_COONa), acetic acid (CH_3_COOH), hydrochloric acid (HCl), potassium persulfate (K_2_S_2_O_8_), sodium chloride (NaCl), monosodium phosphate (NaH_2_PO_4_), and disodium phosphate (Na_2_HPO_4_) were purchased from RCI Labscan Co., Ltd. (Bangkok, Thailand). Bovine serum albumin, AR grade ethanol, and HPLC grade acetonitrile were purchased from Merck (Darmstadt, Germany).

### 3.3. Preparation of M. alba Leaf Extract

#### 3.3.1. Maceration Method

Dried powder of *M*. *alba* leaves was macerated in 95% *v*/*v* ethanol at a weight ratio of 1:5 for 3 cycles of 24 h each at room temperature. The *M*. *alba* leaf residue was removed by filtering through Whatman No. 1 filter paper. The solvent was subsequently removed from the collected filtrate by a rotary evaporator (Eyela, Tokyo, Japan) until dry. The extractions were performed in duplicate. The obtained conventional *M*. *alba* leaf extract was then used in further analyses.

#### 3.3.2. PEF Extraction Method

Dried powder of *M*. *alba* leaves was macerated in 95% *v*/*v* ethanol at a weight ratio of 1:5 in a coaxial-cylindrical PEF chamber (20 mm and 60 mm inner and outer electrode diameter, respectively, (Department of Electrical Engineering, Faculty of Engineering, Rajamangala University of Technology Lanna, Chiang Mai, Thailand). Both electrodes were stainless steel, with 300 mL capacity for containing plant materials. The inner electrode had 20 kV for positive unipolar-exponential decay type, while the outer electrode was connected to ground. The PEF high voltage (20 kV) was generated from the fly back circuit, which gets energy from the direct current (DC) switching power supply of 24 V and 200 W. This high voltage was accumulated in the high-voltage capacitor and transmitted to the PEF treatment chamber by the rotating gap (Department of Electrical Engineering, Faculty of Engineering, Rajamangala University of Technology Lanna, Chiang Mai, Thailand). PEF treatment of 10 kV/cm was applied to the macerate at a frequency of 5 Hz with a pulse width of 1 µs for 20 min, which had a total power consumption of about 0.6 kWh. The macerate was then filtered through Whatman No.1 filter paper. The solvent was subsequently removed from the collected filtrate by using a freeze dryer (Christ Beta 2–8 Ldplus, Martin Christ, Germany). The extractions were performed in triplicate. The obtained PEF *M*. *alba* leaf extract was then used in further analyses.

### 3.4. Determining Chemical Composition of M. alba Leaf Extract

#### 3.4.1. Determining 1-Deoxynojirimycin Content by High-Performance Liquid Chromatography (HPLC)

HPLC analysis was performed using an HP-1100 system (Hewlett Packard, Palo Alto, CA, USA). A reversed phase column (Eclipse XDB-C18, 150 mm × 4.6 mm id, 5 μm; Agilent, Palo Alto, CA, USA) was connected with a guard column (Eclipse XDB-C18, 4.0 mm × 4.6 mm id, 5 μm; Agilent, Palo Alto, CA, USA). An isocratic mobile phase system composed of acetonitrile and 0.1% *v*/*v* trifluoroacetic acid at a ratio of 45:55 was used to elute the sample at a flow rate of 1 mL/min. All samples were detected for UV absorbance (Hewlett Packard, Palo Alto, CA, USA) at 254 nm. 1-Deoxynojirimycin was used as a marker for quantitative analysis. All experiments were performed in triplicate.

#### 3.4.2. Determining Total Phenolic Content

Total phenolic content of each extract was investigated by the Folin–Ciocalteu method as previously described by Chaiyana et al. (2017) [52]. Briefly, 180 μL of 1:10 diluted Folin–Ciocalteu reagent was added to 20 μL of extract in a flat-bottomed 96-well UV plate (Costar, Corning Ltd., Sunderland, UK) and incubated at room temperature in the dark for 4 min. Subsequently, 80 μL of 0.7 M sodium carbonate solution was added, and it was further incubated at room temperature for 2 h. The UV absorbance of the resulting mixture was measured at 750 nm using a multimode detector (Beckman Coulter DTX880, Fullerton, CA, USA). The standard curve was constructed using gallic acid (R^2^ = 0.995). The concentration of phenolic compound, expressed as milligrams of gallic acid equivalent (GAE) per gram of extract, was calculated using Equation (1):

(1) $$\mathrm{Total}\;\mathrm{phenolic}\;\mathrm{content}\;\left(\mathrm{mg}\;\mathrm{GAE}\;\mathrm{per}\;g\;\mathrm{extract}\right)=\frac{\left(A-B\right)-0.0095}{0.0061}$$

where *A* is absorbance of the mixture containing 20 μL of sample solution, 180 μL of Folin–Ciocalteu reagent, and 80 μL of sodium carbonate solution, and *B* is absorbance of the mixture containing 20 μL of sample solution, 80 μL of sodium carbonate solution, and 180 μL of DI water. Three independent experiments repeated in triplicate were performed.

### 3.5. Determining Antioxidant Activity

#### 3.5.1. 2,2′-Diphenyl-1-picrylhydrazyl-hydrate (DPPH) Assay

Scavenging activity against DPPH radicals (DPPH^•^) of each extract was investigated using a DPPH assay according to a method previously described by Chaiyana et al. (2017) [52]. Briefly, 180 μL of 167 μM DPPH solution was added to 20 μL of extract in a flat-bottomed 96-well UV plate (Costar, Corning Ltd., Sunderland, UK) and incubated at room temperature in the dark for 30 min. The UV absorbance of the resulting mixture was measured at 520 nm using a multimode detector (Beckman Coulter DTX880, Fullerton, CA, USA). The DPPH^•^ scavenging activity was calculated using Equation (2):

(2) $${\%\;\mathrm{DPPH}}^{}\;\mathrm{scavenging}\;\mathrm{activity}=\left\{\frac{\left(A-B\right)-\left(C-D\right)}{A-B}\right\}\;\;100$$

where *A* is UV absorbance of a mixture containing 20 μL of DI water and 180 μL of 167 μM DPPH solution, *B* is UV absorbance of a mixture containing 20 μL of DI water and 180 μL of absolute ethanol, *C* is UV absorbance of a mixture containing 20 μL of extract and 180 μL of 167 μM DPPH solution, and *D* is UV absorbance of a mixture containing 20 μL of extract and 180 μL of absolute ethanol. l-ascorbic acid was used as a positive control. Three independent experiments repeated in triplicate were performed.

#### 3.5.2. 2,2′-Azinobis 3-ethylbenzothiazoline-6-sulphonate (ABTS) Assay

Scavenging activity against ABTS radicals (ABTS^•+^) of each extract was investigated using an ABTS assay according to a method previously described by Chaiyana et al. (2017) [52]. Briefly, 3 mL of 2.45 mM potassium persulfate solution was mixed with 2 mL of 7 mM ABTS solution and incubated in the dark for 24 h. The resulting ABTS^•+^ solution was then diluted 20 times with ethanol. Then 180 μL of the ABTS^•+^ solution was added to 20 μL of the sample solution in a flat-bottomed 96-well UV plate (Costar, Corning Ltd., Sunderland, UK) and incubated at room temperature for 5 min. The UV absorbance of the resulting mixture was measured at 750 nm using a microplate reader (Spectrostar Nano, BMG Labtech GmbH, Ortenberg, Germany). The standard curve was constructed using Trolox (R^2^ = 0.998). ABTS^•+^ scavenging activity was expressed as Trolox equivalent antioxidant capacity (TEAC), representing the amount of Trolox (mg) equivalent per g of sample. TEAC was calculated using Equation (3):

(3) $$\mathrm{TEAC}=\frac{\left(A-B\right)-0.7615}{-0.0613}$$

where *A* is absorbance of the mixture containing 20 μL of sample solution and 180 μL of ABTS^•+^ solution and *B* is absorbance of the mixture containing 20 μL of sample solution and 180 μL of DI water. l-ascorbic acid was used as a positive control. Three independent experiments repeated in triplicate were performed.

#### 3.5.3. Ferric Reducing Antioxidant Power (FRAP) Assay

Ferric reducing antioxidant power of each extract was investigated using FRAP assay according to a method previously described by Chaiyana et al. (2017), which had been modified from Saeio et al. (2011) [52,53]. Briefly, FRAP solution was freshly prepared by mixing 10 mL of 0.3 M acetate buffer at pH 3.6 with 1 mL of 10 mM 2,4,6 tripyridyl-s-triazine (TPTZ) solution in 40 mM HCl and 1 mL of 20 mM ferric chloride. Subsequently, 180 μL of the resulting FRAP solution was added to 20 μL of each extract in a flat-bottomed 96-well UV plate (Costar, Corning Ltd., Sunderland, UK) and incubated at room temperature in the dark for 5 min. The UV absorbance of the resulting mixture was measured at 595 nm using a multimode detector (Beckman Coulter DTX880, Fullerton, CA, USA). The standard curve was constructed using FeSO_4_ (R^2^ = 0.999). Ferric reducing power was expressed as equivalent capacity (EC_1_), representing the amount (μM) of FeSO_4_ equivalent per g of sample. EC_1_ was calculated using Equation (4):

(4) $${\mathrm{EC}}_{1}=\frac{\left(A-B\right)-0.0167}{0.1397}$$

where *A* is absorbance of the mixture containing 20 μL of sample solution and 180 μL of FRAP solution and *B* is absorbance of the mixture containing 20 μL of sample solution and 180 μL of DI water. l-ascorbic acid was used as a positive control. Three independent experiments repeated in triplicate were performed.

### 3.6. Determining Anti-Tyrosinase Activity

Each enzyme’s inhibitory activity against tyrosinase was investigated using a spectrophotometric assay according to a method previously described by Laosirisathian et al. (2020), which had been modified from Pomerantz (1976) [54]. l-tyrosine and l-DOPA were used as substrates for the tyrosinase. Briefly, 90 μL of 85 units/mL tyrosinase enzyme dissolved in PBS at pH 6.8 was added to 10 μL of extract in a flat-bottomed 96-well UV plate (Costar, Corning Ltd., Sunderland, UK) and incubated at room temperature for 10 min. Subsequently, 100 μL of 2.5 mM l-tyrosine or l-DOPA was added, and it was incubated at room temperature for a further 30 min. The UV absorbance of the resulting mixture was measured at 450 nm using a multimode detector (Beckman Coulter DTX880, Fullerton, CA, USA). The anti-tyrosinase activity was calculated using Equation (5):

(5) $$\%\;\mathrm{Antityrosinase}\;\mathrm{activity}=\left\{\frac{\left(A-B\right)-\left(C-D\right)}{A-B}\right\}\;\;100$$

where *A* is UV absorbance of a mixture containing 10 μL of DI water, 90 μL of tyrosinase enzyme, and 100 μL of substrate; *B* is UV absorbance of a mixture containing 10 μL of DI water and 190 μL of PBS at pH 6.8; *C* is UV absorbance of a mixture containing 10 μL of extract, 90 μL of tyrosinase enzyme, and 100 μL of substrate; and *D* is UV absorbance of a mixture containing 10 μL of extract and 190 μL of PBS at pH 6.8. Kojic acid was used as a positive control. Various concentrations of sample, ranging from 6.25 to 100 μg/mL, were investigated for percentage of tyrosinase inhibitory activity. Dose response curves of each sample were plot. IC_50_ value, which is a concentration at 50% tyrosinase inhibition, was then calculated using GraphPad Prism (version 2.01, GraphPad Software, San Diego, CA, USA). Three independent experiments repeated in triplicate were performed.

### 3.7. Determining Anti-Hyaluronidase Activity

The hyaluronidase inhibitory activity of each extract was investigated using spectrophotometric assay according to a method previously described by Chaiyana et al. (2019) with slight modifications [55]. First, 15 units/mL of hyaluronidase solution was prepared by dissolving hyaluronidase from bovine testes in 20 mM phosphate buffer at pH 5.35 containing 0.01% *w*/*v* BSA and 77 mM NaCl. Subsequently, 100 µL of hyaluronidase solution was added to 20 µL of extract in a flat-bottomed 96-well UV plate (Costar, Corning Ltd., Sunderland, UK) and incubated at 37 °C for 10 min. Subsequently, 100 µL of 0.03% *w*/*v* hyaluronic acid in phosphate buffer at pH 5.35 was added, and it was further incubated at 37 °C for 45 min. The UV absorbance of the resulting mixture was measured at 600 nm using a multimode detector (Beckman Coulter DTX880, Fullerton, CA, USA). The hyaluronidase inhibitory activity was calculated using Equation (6):

(6) $$\%\;\mathrm{Hyaluronidase}\;\mathrm{activity}=\left\{\frac{\left(A-B\right)-\left(C-D\right)}{A-B}\right\}\;\;100$$

where *A* is UV absorbance of a mixture containing 20 µL of DI water, 100 µL of hyaluronidase solution, and 100 µL of hyaluronic acid solution; *B* is UV absorbance of a mixture containing 20 µL of DI water and 200 µL of phosphate buffer at pH 5.35; *C* is UV absorbance of a mixture containing 20 µL of extract, 100 µL of hyaluronidase solution, and 100 µL of hyaluronic acid solution; and *C* is UV absorbance of a mixture containing 20 µL of extract and 200 µL of phosphate buffer at pH 5.35. Oleanolic acid was used as a positive control. Three independent experiments repeated in triplicate were performed.

### 3.8. Statistical Analysis

All data are presented as a mean ± standard deviation (SD). Statistical significance was analyzed by one-way analysis of variance (ANOVA) followed by Tukey’s post-hoc test using SPSS 17.0 for Windows (SPSS Inc., Chicago, IL, USA). *P* < 0.05 was considered statistically significant.

## 4. Conclusions

The present study is the first to highlight PEF as an extraction method capable of enhancing phenolic content in *M. alba* leaf extract. In addition, PEF significantly enhanced antioxidant, anti-tyrosinase, and anti-hyaluronidase activity of *M. alba* leaf extract (*P* < 0.05). Although there are many articles dealing with PEF extraction, most of them are aimed at using PEF in the food industry. There are only a few studies dealing with cosmetic applications. The present study is hence the first to reveal the potential of PEF extraction to enhance the variety of cosmetic activities of *M. alba* leaves for use in the cosmetic industry. However, the location where *M. alba* leaves were collected had an effect on their biological activity. PEF extract of leaves collected from BR contained the highest phenolic content and also exhibited the highest radical scavenging activity, ferric reducing antioxidant power, and anti-tyrosinase activity (*P* < 0.05). Its ABTS^•+^ scavenging activity was comparable to that of l-ascorbic acid and its anti-tyrosinase activity was comparable to that of kojic acid (*P* > 0.05). In addition, this study is also the first to report anti-hyaluronidase activity of *M. alba* leaves. The PEF extract of leaves collected from SK had the highest anti-hyaluronidase activity, which was comparable to that of oleanolic acid (*P* > 0.05). PEF not only could enhance the variety of cosmetic activities of *M. alba* leaves, but also is a green extraction method, which could minimize the consumption of organic solvent and energy and the duration of extraction. Therefore, PEF is suggested for further use for the production of *M. alba* leaf extract, which is an attractive natural whitening and anti-aging agent. Additionally, a further microscopic study is suggested to understand the mechanism and effects of PEF extraction.

## References

[1] Yuan Q., Zhao L. (2017). The Mulberry (*Morus alba* L.) Fruit—A Review of Characteristic Components and Health Benefits. J. Agric. Food Chem..

[2] Devi B., Sharma N., Kumar D., Jeet K. (2013). *Morus alba* Linn: A phytopharmacological review. Int. J. Pharm. Pharm. Sci..

[3] Qin C., Li Y., Niu W., Ding Y., Zhang R., Shang X. (2010). Analysis and characterisation of anthocyanins in mulberry fruit. Czech J. Food Sci..

[4] Khamenei-Tabrizi A.S., Sendi J.J., Imaani S., Shojaee M. (2020). Can Feeding of Silkworm on Different Mulberry Variety Affect Its Performance?. J. Econ. Entomol..

[5] Chen G.H., Tong J.J., Wang F., Hu X.Q., Li X.W., Tao F., Wei Z.J. (2015). Chronic adjunction of 1-deoxynojirimycin protects from age-related behavioral and biochemical changes in the SAMP8 mice. Age.

[6] Wang R.J., Yang C.H., Hu M.L. (2010). 1-Deoxynojirimycin inhibits metastasis of B16F10 melanoma cells by attenuating the activity and expression of matrix metalloproteinases-2 and-9 and altering cell surface glycosylation. J. Agric. Food Chem..

[7] Jeanmaire C., Danoux L., Pauly G. (2001). Glycation during human dermal intrinsic and actinic ageing: An in vivo and in vitro model study. Br. J. Derm..

[8] Chang L.W., Juang L.J., Wang B.S., Wang M.Y., Tai H.M., Hung W.J., Chen Y.J., Huang M.H. (2011). Antioxidant and antityrosinase activity of mulberry (*Morus alba* L.) twigs and root bark. Food Chem. Toxicol..

[9] Iqbal S., Younas U., Chan K.W., Sarfraz R.A., Uddin M. (2012). Proximate composition and antioxidant potential of leaves from three varieties of Mulberry (*Morus* sp.): A comparative study. Int. J. Mol. Sci..

[10] Park S., Kim Y.S., Lee H.A., Lim Y., Kim Y. (2013). Mulberry Leaf Extract Inhibits Invasive Potential and Downregulates Hypoxia-Inducible Factor-1α (HIF-1α) in SK-N-BE (2) C Neuroblastoma Cells. Biosci. Biotechnol. Biochem..

[11] Nowacka M., Tappi S., Wiktor A., Rybak K., Miszczykowska A., Czyzewski J., Drozdzal K., Witrowa-Rajchert D., Tylewicz U. (2019). The Impact of Pulsed Electric Field on the Extraction of Bioactive Compounds from Beetroot. Foods.

[12] Mannozzi C., Rompoonpol K., Fauster T., Tylewicz U., Romani S., Dalla Rosa M., Jaeger H. (2019). Influence of Pulsed Electric Field and Ohmic Heating Pretreatments on Enzyme and Antioxidant Activity of Fruit and Vegetable Juices. Foods.

[13] Zbinden M.D.A., Sturm B.S., Nord R.D., Carey W.J., Moore D., Shinogle H., Stagg-Williams S.M. (2013). Pulsed electric field (PEF) as an intensification pretreatment for greener solvent lipid extraction from microalgae. Biotechnol. Bioeng..

[14] Parniakov O., Lebovka N.I., Van Hecke E., Vorobiev E. (2014). Pulsed electric field assisted pressure extraction and solvent extraction from mushroom (*Agaricus bisporus*). Food Bioprocess Tech..

[15] Bozinou E., Karageorgou I., Batra G., Dourtoglou V.G., Lalas S.I. (2019). Pulsed electric field extraction and antioxidant activity determination of *Moringa oleifera* dry leaves: A comparative study with other extraction techniques. Beverages.

[16] Ricci A., Parpinello G.P., Versari A. (2018). Recent advances and applications of pulsed electric fields (PEF) to improve polyphenol extraction and color release during red winemaking. Beverages.

[17] Poojary M.M., Barba F.J., Aliakbarian B., Donsì F., Pataro G., Dias D.A., Juliano P. (2016). Innovative alternative technologies to extract carotenoids from microalgae and seaweeds. Mar. Drugs..

[18] Grimi N., Dubois A., Marchal L., Jubeau S., Lebovka N.I., Vorobiev E. (2014). Selective extraction from microalgae *Nannochloropsis* sp. using different methods of cell disruption. Bioresour. Technol..

[19] Luengo E., Martínez J.M., Bordetas A., Álvarez I., Raso J. (2015). Influence of the treatment medium temperature on lutein extraction assisted by pulsed electric fields from *Chlorella vulgaris*. Innov. Food Sci. Emerg. Technol..

[20] Luengo E., Condón-Abanto S., Álvarez I., Raso J. (2014). Effect of pulsed electric field treatments on permeabilization and extraction of pigments from *Chlorella vulgaris*. J. Membr. Biol..

[21] Parniakov O., Barba F.J., Grimi N., Marchal L., Jubeau S., Lebovka N., Vorobiev E. (2015). Pulsed electric field and pH assisted selective extraction of intracellular components from microalgae *Nannochloropsis*. Algal. Res..

[22] Grosso C., Valentão P., Ferreres F., Andrade P.B. (2015). Alternative and efficient extraction methods for marine-derived compounds. Mar. Drugs..

[23] Poojary M.M., Lund M.N., Barba F.J., Francisco J.B., Oleksii P., Artur W. (2020). Pulsed electric field (PEF) as an efficient technology for food additives and nutraceuticals development. Pulsed Electric Fields to Obtain Healthier and Sustainable Food for Tomorrow.

[24] Barba F.J., Roselló-Soto E., Marszałek K., Kovačević D.B., Jambrak A.R., Lorenzo J.M., Chemat F., Putnik P., Chemat F., Vorobiev E. (2019). Green food processing: Concepts, strategies, and tools. Green Food Processing Techniques.

[25] Prabhu M.S., Levkov K., Livney Y.D., Israel A., Golberg A. (2019). High-Voltage Pulsed Electric Field Preprocessing Enhances Extraction of Starch, Proteins, and Ash from Marine Macroalgae *Ulva ohnoi*. Acs Sustain. Chem. Eng..

[26] Boussetta N., Vorobiev E., Le L.H., Cordin-Falcimaigne A., Lanoisellé J.L. (2012). Application of electrical treatments in alcoholic solvent for polyphenols extraction from grape seeds. LWT-Food Sci. Technol..

[27] Yatsunami K., Ichida M., Onodera S. (2008). The relationship between 1-deoxynojirimycin content and α-glucosidase inhibitory activity in leaves of 276 mulberry cultivars (*Morus* spp.) in Kyoto, Japan. J. Nat. Med..

[28] Hu X.Q., Jiang L., Zhang J.G., Deng W., Wang H.L., Wei Z.J. (2013). Quantitative determination of 1-deoxynojirimycin in mulberry leaves from 132 varieties. Ind. Crop. Prod..

[29] Boonprong M. (2014). Public policy with promotion to Thai swamp buffaloes production: A case study on raising Thai swamp buffaloes and satisfaction of local farmers in Buriram province, Thailand. AFBE 2014 conference paper, Proceedings of AFBE 2014 conference paper, Thaksin University, Thailand, 5–6 November 2014.

[30] Sriprom M., Chalvet-Monfray K., Chaimane T., Vongsawat K., Bicout D.J. (2010). Monthly district level risk of dengue occurrences in Sakon Nakhon Province, Thailand. Sci. Total Env..

[31] Doi R., Itoh M., Chakhatrakan S., Uga S. (2016). Epidemiological investigation of parasitic infection of schoolchildren from six elementary schools in Sakon Nakhon Province, Thailand. Kobe J. Med. Sci..

[32] Tangtrongsup S., Scorza A.V., Reif J.S., Ballweber L.R., Lappin M.R., Salman M.D. (2020). Seasonal distributions and other risk factors for *Giardia duodenalis* and *Cryptosporidium* spp. infections in dogs and cats in Chiang Mai, Thailand. Prev. Vet. Med..

[33] Agcam E., Akyıldız A., Evrendilek G.A. (2014). Comparison of phenolic compounds of orange juice processed by pulsed electric fields (PEF) and conventional thermal pasteurisation. Food Chem..

[34] López-Giral N., González-Arenzana L., González-Ferrero C., López R., Santamaría P., López-Alfaro I., Garde-Cerdán T. (2015). Pulsed electric field treatment to improve the phenolic compound extraction from Graciano, Tempranillo and Grenache grape varieties during two vintages. Innov. Food Sci. Emerg. Technol..

[35] Lohani U.C., Muthukumarappan K. (2016). Application of the pulsed electric field to release bound phenolics in sorghum flour and apple pomace. Innov. Food Sci. Emerg. Technol..

[36] Liu Z.W., Zeng X.A., Ngadi M. (2018). Enhanced extraction of phenolic compounds from onion by pulsed electric field (PEF). J. Food Process. Pres..

[37] Fincan M. (2015). Extractability of phenolics from spearmint treated with pulsed electric field. J. Food Eng..

[38] Bhat Z.F., Morton J.D., Mason S.L., Bekhit A.E.D.A. (2019). Current and future prospects for the use of pulsed electric field in the meat industry. Crit. Rev. Food Sci. Nutr..

[39] Wiktor A., Singh A.P., Parniakov O., Mykhailyk V., Mandal R., Witrowa-Rajchert D., Francisco J.B., Oleksii P., Artur W. (2020). PEF as an alternative tool to prevent thermolabile compound degradation during dehydration processes. Pulsed Electric Fields to Obtain Healthier and Sustainable Food for Tomorrow.

[40] Brochier B., Mercali G.D., Marczak L.D.F. (2019). Effect of moderate electric field on peroxidase activity, phenolic compounds and color during ohmic heating of sugarcane juice. J. Food Process. Preserv..

[41] Li Y.G., Ji D.F., Zhong S., Lv Z.Q., Lin T.B., Chen S., Hu G.Y. (2011). Hybrid of 1-deoxynojirimycin and polysaccharide from mulberry leaves treat diabetes mellitus by activating PDX-1/insulin-1 signaling pathway and regulating the expression of glucokinase, phosphoenolpyruvate carboxykinase and glucose-6-phosphatase in alloxan-induced diabetic mice. J. Ethnopharmacol..

[42] Przybylska-Balcerek A., Stuper-Szablewska K. (2019). Phenolic acids used in the cosmetics industry as natural antioxidants. EJMT.

[43] Shahidi F., Ambigaipalan P. (2015). Phenolics and polyphenolics in foods, beverages and spices: Antioxidant activity and health effects–A review. J. Funct. Foods..

[44] Poljšak B., Dahmane R. (2012). Free radicals and extrinsic skin aging. Derm. Res. Pr..

[45] Rinnerthaler M., Bischof J., Streubel M.K., Trost A., Richter K. (2015). Oxidative stress in aging human skin. Biomolecules.

[46] Winterbourn C.C. (2020). Biological chemistry of superoxide radicals. Chemtexts.

[47] Igielska-Kalwat J., Gościańska J., Nowak I. (2015). Carotenoids as natural antioxidants. Post. Hig. Med. Dosw..

[48] Silva S., Ferreira M., Oliveira A.S., Magalhaes C., Sousa M.E., Pinto M., Sousa Lobo J.M., Almeida I.F. (2019). Evolution of the use of antioxidants in anti-ageing cosmetics. Int. J. Cos. Sci..

[49] Hanh N.T.M., Phung N.K.P., Phuong Q.N.D. (2017). Studying on Tyrosinase Inhibition Activity of Some Vietnamese Folk Plants Aims to Use in Skin-Whitening Cosmetics. Am. J. Plant Sci..

[50] Couteau C., Coiffard L. (2016). Overview of skin whitening agents: Drugs and cosmetic products. Cosmetics.

[51] Buhren B.A., Schrumpf H., Hoff N.P., Bölke E., Hilton S., Gerber P.A. (2016). Hyaluronidase: From clinical applications to molecular and cellular mechanisms. Eur. J. Med. Res..

[52] Chaiyana W., Punyoyai C., Somwongin S., Leelapornpisid P., Ingkaninan K., Waranuch N., Srivilai J., Thitipramote N., Wisuitiprot W., Schuster R. (2017). Inhibition of 5α-reductase, IL-6 secretion, and oxidation process of *Equisetum debile* Roxb. ex vaucher extract as functional food and nutraceuticals ingredients. Nutrients.

[53] Saeio K., Chaiyana W., Okonogi S. (2011). Antityrosinase and antioxidant activities of essential oils of edible Thai plants. Drug Discov..

[54] Laosirisathian N., Saenjum C., Sirithunyalug J., Eitssayeam S., Sirithunyalug B., Chaiyana W. (2020). The Chemical Composition, Antioxidant and Anti-Tyrosinase Activities, and Irritation Properties of Sripanya *Punica granatum* Peel Extract. Cosmetics.

[55] Chaiyana W., Anuchapreeda S., Punyoyai C., Neimkhum W., Lee K.H., Lin W.C., Lue S.C., Viernstein H., Mueller M. (2019). *Ocimum sanctum* Linn. as a natural source of skin anti-ageing compounds. Ind. Crops Prod..

